# Fluctuation and diversity of Hydromedusae (Hydrozoa, Cnidaria) in a highly productive region of the Gulf of Mexico inferred from high frequency plankton sampling

**DOI:** 10.7717/peerj.7848

**Published:** 2019-10-08

**Authors:** Sarah Pruski, Maria Pia Miglietta

**Affiliations:** Department of Marine Biology, Texas A&M University—Galveston, Galveston, TX, United States of America

**Keywords:** Hydrozoa, Medusa, Jellyfish, Bloom, Temperature, Galveston, Gulf of Mexico, Molecular Barcoding

## Abstract

Hydrozoa medusae undergo blooms and seasonal fluctuations; however the drivers of such fluctuations are unknown. To understand how medusa populations fluctuate in response to seasonal factors such as temperature, salinity, dissolved oxygen, and chlorophyll a, and to enhance our taxonomic knowledge of Hydrozoa in Galveston Bay (TX), we performed frequent plankton sampling from September 2015 to September 2016. We collected 1,321 medusae in 190 sampling days. Using molecular barcoding and morphological analyses we identified 25 species, of which 21 are a first record for Galveston Bay and eight for the Gulf of Mexico. Daily medusa abundance is non-linearly related to temperature, with peak abundance estimated with multivariate regression analysis at approximately 21C. The role that temperature plays in driving medusa abundance has implications for future climate change scenarios, given that temperature in the Gulf of Mexico is expected to rise 4 °C by the end of the century. We also show that the biodiversity of the Galveston Bay and the Gulf of Mexico is underestimated and that molecular barcoding is an important and efficient tool to identify large number of medusae. We conclude that dense plankton sampling is necessary to capture both diversity and abundance of planktonic medusae.

## Introduction

Jellyfish are top predators, feed on zooplankton, and compete with economically important fish (Mills, 2001; Coma et al., 2000; Richardson et al., 2009; Purcell, 1991; Wintzer, Meek & Moyle, 2011). They also fluctuate seasonally and undergo massive, short-lived blooms (Boero et al., 2008). While the term “jellyfish” usually refers to the Cnidarian classes of Scyphozoa, Hydrozoa, and Cubozoa and the phylum Ctenophora, the medusozoans (Scyphozoa, Hydrozoa, and Cubozoa) make up the majority (in terms of abundance and diversity) of gelatinous zooplankton (Collins, 2002; Mills, 2001). Within the phylum Cnidaria, the Hydrozoa is the most diverse, widespread, and least studied class with medusae (jellyfish).

Medusae are seasonally produced in a synchronized fashion by the benthic polyps (Boero & Bouillon, 1993), but the cues triggering medusa production (and therefore their blooms) are unknown in most species. Circannual rhythms, temperature, salinity, moon phases, dissolved oxygen, and water turbidity have all been proposed as possible cues, but have only been tested on few individual species (Brock, 1975; Genzano & Kubota, 2003; Ma & Purcell, 2005; Stefani, 1956; Werner, 1954; Werner, 1961; Nowaczyk et al., 2016; Wintzer, Meek & Moyle, 2013). Upwelling, which often correlated with high productivity, has also been linked to medusa blooms in tropical waters (Miglietta, Rossi & Collin, 2008). However, a comprehensive understanding of the conditions that result in Hydrozoa synchronized medusa budding and thus blooms, is at the moment lacking.

Studies on medusa blooms often display sampling that is geographically broad (i.e., several sampling locations) but rarely frequent enough to capture the fast changes that are typical of plankton populations. Peak of medusa abundance, for example, are short lived (Miglietta, Rossi & Collin, 2008), thus seasonal or monthly sampling may fail to detect days of high abundance and will capture only partial biodiversity, as rare species, or species with very short blooms may remain unsampled. Moreover, with more than 3,800 nominal species (http://www.marinespecies.org/hydrozoa), proper sorting and identification of Hydrozoa medusae is time consuming, and requires massive taxonomic expertise (Zheng et al., 2014). Identification of Hydrozoa medusae is further hindered by their small size, phenotypic plasticity, and the presence of cryptic species (Calder & Cairns, 2009; Zheng et al., 2014; Miglietta, Schuchert & Cunningham, 2009; Miglietta, Maggioni & Matsumoto, 2018). Regular frequent sampling of Hydromedusae thus comes with a considerable taxonomic burden that may be alleviated by DNA barcoding techniques. The 5′ region of mitochondrial cytochrome c oxidase subunit I (COI) is the standard barcoding marker for most animals (Moura et al., 2011). Although there has been some success using COI to barcode Hydrozoa (Bucklin, Steinke & Blanco-Bercial, 2011; Govindarajan, Halanych & Cunningham, 2005), the large ribosomal subunit of the mitochondrial RNA (lsu-rRNA, 16S) is easier to amplify and an excellent low-cost tool to identify species in Hydrozoa (Miglietta, 2016; Miglietta et al., 2015; Zheng et al., 2014). It has been used in a wide range of studies for accurate determination of species diversity and taxonomic revision within the Hydrozoa (Moura et al., 2018; Schuchert, 2014); but see (Schuchert, 2018) for exceptions), and it is widely considered the barcoding molecule for Hydrozoa.

In this paper we analyze the Hydrozoa biodiversity of the Galveston Bay, a notorious fishing ground on the Texan coast of the Gulf of Mexico (GoM) and the seventh largest estuary in the United States, using high frequency sampling and molecular barcoding techniques. More specifically we aim to (a) assess the diversity of the medusae of the class Hydrozoa in the Galveston Bay, using a combination of morphological and molecular barcoding tools; (b) characterize their seasonality and their blooms through high frequency sampling across a year, and (c) investigate the role of abiotic factors such as temperature, salinity and primary productivity in driving seasonality and bloom occurrence. The ultimate goal of this paper is to contribute to the understanding of patterns of plankton and its variability, as they yield important insights into the forces structuring the ecosystem.

## Methods

### Medusa collection

**Figure 1:**
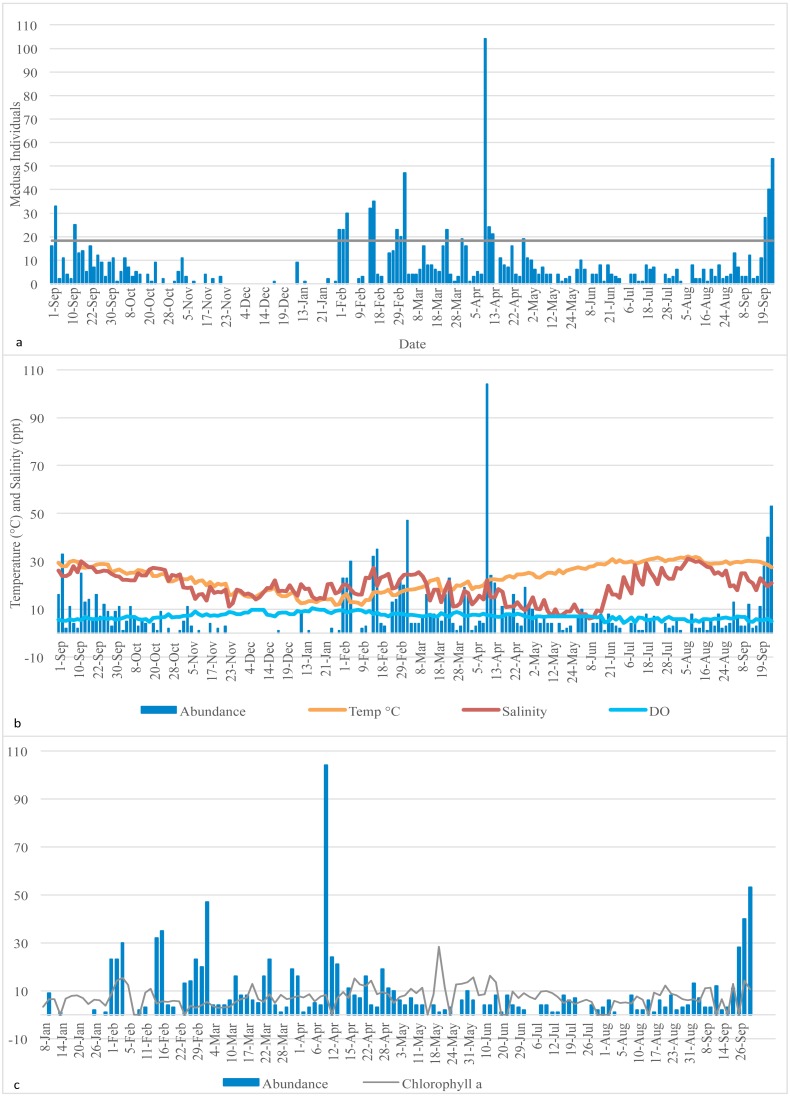
Medusa abundance. (A) Medusa abundance per sampling day. The dashed line represents one standard deviation above the mean abundance. (B) Daily temperature (° C), salinity (ppt), and dissolved oxygen (DO) (mg/L) against the daily medusa abundance. (C) Daily medusa abundance against daily chlorophyll a (µg/L). Note: Chlorophyll a data are available from January to September 2016 only.

Planktonic samples were collected, using a 100-micron net, 90 cm long, and a 30-cm mouth. The small mesh size prevented damage to any delicate hydromedusae that were collected. Plankton tows were conducted within the boat basin at Texas A&M University Galveston on Pelican Island (29°18′47.0′N 94°48′59.8′W). The basin receives seawater from Galveston Bay through the ship channel. Two tows per day were conducted three to four times a week from September 2015 to September 2016. The samples were collected during the morning, and each tow consisted of towing the net six times along the side of the dock by walking back and forth at a constant rate for a total of 156 m. The net was kept completely submerged in the water during the tows, to ensure that approximately the same amount of water was filtered for each sample. The plankton collected during the two consecutive tows were combined and considered as a single daily sample, and examined in the laboratory under a Leica M80 Stereomicroscope. Hydromedusae were isolated from other planktonic organisms using a pipette, counted and photographed using a Leica M80 Stereomicroscope connected to a Leica MC170 HD camera. Daily counts of medusae are shown in Fig. 1 and Table S1. Medusae were morphologically identified to the lowest possible taxonomic level using appropriate taxonomic keys (e.g., Bouillon et al., 2006) and preserved in ethanol for molecular analysis. The number of species and total hydromedusae abundance (i.e., individual count) of each sample was recorded.

### Total medusa abundance and correlation with abiotic factors

Daily temperature (^∘^C), salinity (ppt), dissolved oxygen (mg/L) (DO), and chlorophyll a (µg/L) of the Galveston Bay were made available by the Phytoplankton Dynamics Laboratory at Texas A&M University at Galveston. Temperature, salinity and DO were measured each morning at the same time as the plankton tows occurred; therefore, they reflect the water conditions at the time of sampling. Chlorophyll a data is available from January 2016 to the end of the sampling period only, so statistical analysis involving chlorophyll and medusa abundance was conducted on the January 2016 to September 2016 subsample (raw data are in Table S1). Descriptive statistics of the distribution of medusa abundance and the explanatory variables are reported for both the full sample (Table 1) and four subsamples based on the quartiles of the recorded temperature (Table 2). Medusa daily abundance (number of medusa collected in each sampling day) was plotted against the date of collection. A “bloom” was defined as any day with an abundance at least 1 standard deviation from the mean daily abundance (Miglietta, Rossi & Collin, 2008).

**Table 1:** Statistical description of the variables.

**Variable**	**Mean**	**Median**	**StdDev**	**Min**	**Max**	**N**
*# of Medusae*	6.09	4	11.321	0	104	190
*DO*	7.1094	7	1.3248	4.23	10.42	187
*Salinity*	19.413	19.73	5.8718	6	31.97	188
*Temperature*	23.173	24.21	5.8166	11.7	32	190
*Chlorophyll a*	4.9369	5	2.7112	0	9	111

**Table 2:** Statistical description of the variables based on the quartiles of the recorded temperatures.

**Temperature quartile**	**Variables**	**N of days**	**Sum**	**Mean**	**StdDev**	**MIN**	**MAX**
*Q1: low*	# of Medusae	47	260	5.5319	9.8574	0	35
*Q1: low*	Temperature	47	716.87	15.2526	1.9096	11.68	18.05
*Q1: low*	Salinity	47	856.62	18.226	3.4457	10.98	27.3
*Q1: low*	DO	47	411.47	8.7547	0.7889	6.96	10.42
*Q1: low*	Chlorophyll a	28	126	4.5	2.3805	0	9
*Q2: mildly low*	# of Medusae	48	415	8.6458	16.5754	0	104
*Q2: mildly low*	Temperature	48	1004.62	20.9296	1.7252	18.13	23.86
*Q2: mildly low*	Salinity	48	842.51	17.5523	4.5023	10	25.42
*Q2: mildly low*	DO	48	358.3	7.4646	0.5259	6.64	9
*Q2: mildly low*	Chlorophyll a	28	148	5.2857	2.5071	1	9
*Q3:mildly high*	# of Medusae	48	401	8.3542	10.4117	0	53
*Q3:mildly high*	Temperature	48	1266.2	26.3792	1.5102	23.92	28.85
*Q3:mildly high*	Salinity	46	826.06	17.9578	7.9634	6	30.06
*Q3:mildly high*	DO	46	293.68	6.3843	0.7546	4.57	8.13
*Q3:mildly high*	Chlorophyll a	21	76	3.619	3.4565	1	9
*Q4: high*	# of Medusae	47	236	5.0213	5.1982	0	28
*Q4: high*	Temperature	47	1415.14	30.1094	0.8691	28.88	31.97
*Q4: high*	Salinity	47	1124.4	23.9234	4.1931	14.66	31.08
*Q4: high*	DO	46	266.01	5.7828	0.6874	4.23	7.08
*Q4: high*	Chlorophyll a	34	198	5.8235	2.3024	1	9

**Figure 2:**
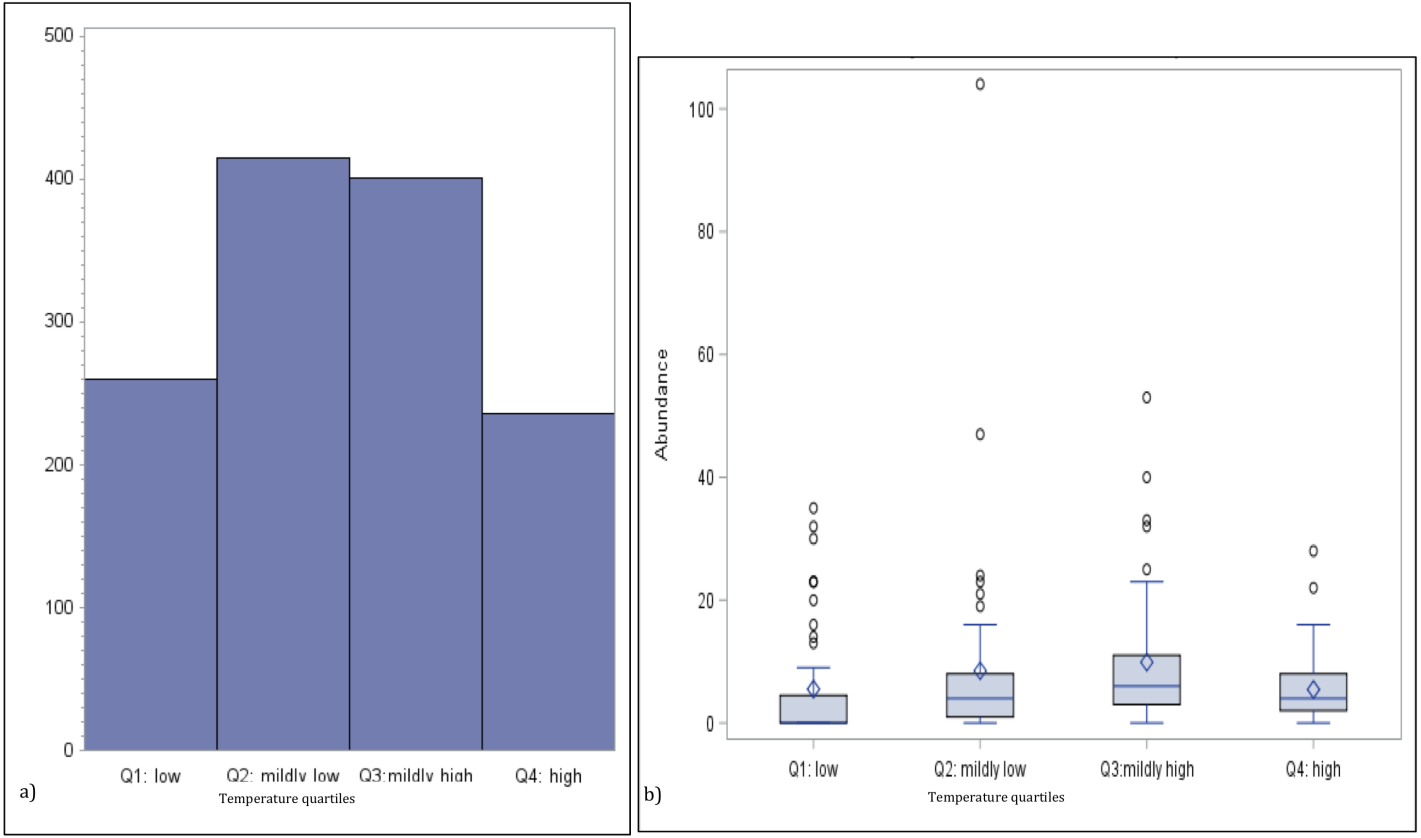
Numbers of medusa grouped for temperature quartiles. (A) Total counts and (B) box plots of numbers of medusa grouped for temperature quartiles (lowest 25%; medium–low 25%; medium–high 25%; high 25%).

To investigate the potential non-linear effect of temperature on jellyfish abundance, we generated a bar chart of the total medusa count by temperature quartiles and a boxplot of the distribution of the abundance for the same temperature quartiles group (see Figs. 2A and Figs. 2B). The univariate analysis linking abundance to potential explanatory variables is concluded with the calculation of their correlation matrix (*p*-values testing the null hypothesis of zero-collation reported in parenthesis) (Table 3).

In addition to performing univariate analysis, we performed multivariate regression analysis based on ordinary least squares (OLS) in SAS. This approach allows for the identification of the effect of any of the explanatory variables on abundance, while controlling for the effect of the other explanatory variables included in the model. The general specification of the regression models is given by

(1) \begin{eqnarray*}{y}_{t}={\alpha }_{t}+{\beta }^{{}^{{^{\prime}}}}{x}_{t}+{\varepsilon }_{t}\end{eqnarray*}

where *t* represent time (calendar day), *y*_*t*_ is abundance at date t, which is measured either in actual counts of medusa (model 1–5) or their natural logarithm (model 6–10), and *x*_*t*_ is a vector of explanatory variables (regressors) linked to medusa abundance (models differ depending on which regressors are included in the vector *x*_*t*_). The use of the natural logarithm of medusa counts is proposed to account for the skewness of the distribution of daily counts and its potential detrimental effect on the statistical fit of the model. The elements of the vector *β*, which we estimate with OLS, capture the effect that the corresponding elements of the regressor vector (*x*_*t*_) have on abundance. *α*_*t*_ is an intercept that is either constant (equal for every t) or varying by quarter (i.e., quarterly time fixed effects) depending on whether the model controls for potential unobserved factors not captured by the proposed explanatory variables. Finally, ε_*t*_ represent an error term uncorrelated with the regressors (*x*_*t*_) that captures the unexplained variation in medusa abundance (Table 4).

**Table 3:** Correlation matrix (with *p*-values) for the entire year. Correlation matrix shows correlation between the variable.

**Variable**	**# of Medusae**	**Temp**	**Salinity**	**DO**	**chloro**	**N of days**
*# of Medusae*	1	0.03299 (0.6513)	0.12112 (0.0978)	−0.09548 (0.1936)	−0.06193 (0.5185)	190
*Temperature*	0.03299 (0.6513)	1	0.30658 (<.0001)	−0.88326 (<.0001)	0.08328 (0.3849)	190
*Salinity*	0.12112 (0.0978)	0.30658 (<.0001)	1	−0.47906 (<.0001)	0.0613 (0.5227)	188
*Dissolved Oxygen*	−0.09548 (0.1936)	−0.88326 (<.0001)	−0.47906 (<.0001)	1	−0.12837 (0.1834)	187
*Chlorophyll a*	−0.06193 (0.5185)	0.08328 (0.3849)	0.0613 (0.5227)	−0.12837 (0.1834)	1	111

**Table 4:** Multivariate analyses.

		Dependent Variable: abundance							Dependent Variable: ln(Abundance+1)					
**Label**	**LABEL OF FORMER VARIABLE**	**Model1**	**Model2**	**Model3**	**Model4**	**Model5**	**Model6**	**Model7**	**Model8**	**Model9**	**Model10**
Temperature	Parameter Estimate	−0.4025	3.2488	6.1668	7.996	.	−0.00232	0.4534	0.865	0.93245	.
	*t* Value	−1.27	2.1627	3.877	3.3479	.	−0.07662	3.20244	6.3196	5.22767	.
	Pr >—t—	0.2057	0.0319	0.0001	0.0011	.	0.93901	0.00161	0	0	.
Squared Temp	Parameter Estimate	.	−0.0771	−0.1498	−0.1821	.	.	−0.00962	−0.0186	−0.02074	.
	*t* Value	.	−2.4851	−4.3244	−3.7525	.	.	−3.29082	−6.2464	−5.72228	.
	Pr >—t—	.	0.0139	0	0.0003	.	.	0.0012	0	0	.
Salinity	Parameter Estimate	0.1422	0.3157	0.6793	0.7011	0.30261	0.00512	0.02678	0.0574	0.05214	0.02393
	*t* Value	0.8405	1.7459	2.873	2.5451	1.67617	0.3172	1.57111	2.8205	2.5345	1.39222
	Pr >—t—	0.4017	0.0825	0.0046	0.0124	0.09544	0.75146	0.1179	0.0053	0.01277	0.16557
Dissolved Oxigen	Parameter Estimate	−2.0878	−0.8277	0.2716	0.9641	−1.80788	−0.22631	−0.06904	0.0601	0.07184	−0.18523
	*t* Value	−1.385	−0.527	0.1832	0.3876	−1.32169	−1.57341	−0.4664	0.4711	0.38673	−1.42224
	Pr >—t—	0.1678	0.5988	0.8549	0.6991	0.18795	0.11736	0.64149	0.6381	0.69976	0.15669
Chlorophyll	Parameter Estimate	.	.	.	−0.2454	.	.	.	.	−0.0467	.
	*t* Value	.	.	.	−0.5312	.	.	.	.	−1.35332	.
	Pr >—t—	.	.	.	0.5964	.	.	.	.	0.17892	.
Q1: low	Parameter Estimate	.	.	.	.	7.74351	.	.	.	.	0.1673
	*t* Value	.	.	.	.	1.78442	.	.	.	.	0.40491
	Pr >—t—	.	.	.	.	0.07604	.	.	.	.	0.68602
Q2: mildly low	Parameter Estimate	.	.	.	.	8.72895	.	.	.	.	0.55673
	*t* Value	.	.	.	.	2.85814	.	.	.	.	1.91455
	Pr >—t—	.	.	.	.	0.00476	.	.	.	.	0.05714
Q3:mildly high	Parameter Estimate	.	.	.	.	6.51577	.	.	.	.	0.59982
	*t* Value	.	.	.	.	2.57619	.	.	.	.	2.49079
	Pr >—t—	.	.	.	.	0.01079	.	.	.	.	0.01365
Adj R-Sq	cValue2	0.97%	3.71%	17.55%	11.66%	4.29%	5.89%	10.71%	36.27%	24.46%	9.43%
N. of Obs		186	186	186	109	186	186	186	186	109	186

### Molecular barcoding

Species identification was confirmed using the hydrozoan barcoding molecule (a ∼600 bp fragment of the large ribosomal subunit of the mitochondrial RNA (lsu-rRNA, 16S)). Genomic DNA was extracted using a protocol modified from Zietara et al. (2000). The lsu-rRNA 16S was amplified using primers SHA (5′ACGGAATGAACTCAAATCATG T-3′) and SHB (5′-TCGACTGTTTACCAAAAACA TA-3′) and the following PCR conditions: 1 min at 94 °C, 35 cycles of 94 °C for 15 s, 50 °C for 1:30 min and 72 °C for 2:30 min, and a final extension at 72 °C for 5 min. PCR products were purified using exoSAP-it following manufacturer protocol. The purified PCR product was run on a 1% agarose gel stained with Sybrsafe at 100 W for 20 min to determine presence/absence of DNA. Confirmed PCR products were sent to the Genomics Core Lab at Texas A&M University Corpus Christi for sequencing analysis.

All sequences were edited in Geneious 10.0.5. Sequences from each species were run through the National Center for Biotechnology Information (NCBI) Basic Local Alignment Search Tool (BLAST) to confirm species identification. For each sequence and its most significant BLAST hit, identity scores and *e*-values were evaluated (see Table S2). Morphological analyses and barcoding and BLAST results were compared for correct species identification.

### Species richness and dominant species

Species diversity was calculated monthly using the Shannon Weaver index. The species dominance index was calculated using Eq. (1) (Wang et al., 2016):

(2) \begin{eqnarray*}Y= \frac{{n}_{1}}{N} {f}_{i}\end{eqnarray*}

where *n* is the number of individual species *i*; *f* is frequency of species *i* throughout the sampling period; *N* is the total number of individuals. Species with a dominance index more than 0.02 were taken as dominant species. The seasonality of five dominant species (*Liriope tetraphylla, Blackfordia virginica, Malagazzia carolinae*, *Clytia gracilis*, and *Nemopsis bachei)* and the dominant genus *Obelia* were further investigated using multivariate regression analyses. SAS University Edition was used to generate regression models to determine correlation with abiotic factors.

## Results

### Time variation of total hydromedusa abundance

**Figure 3:**
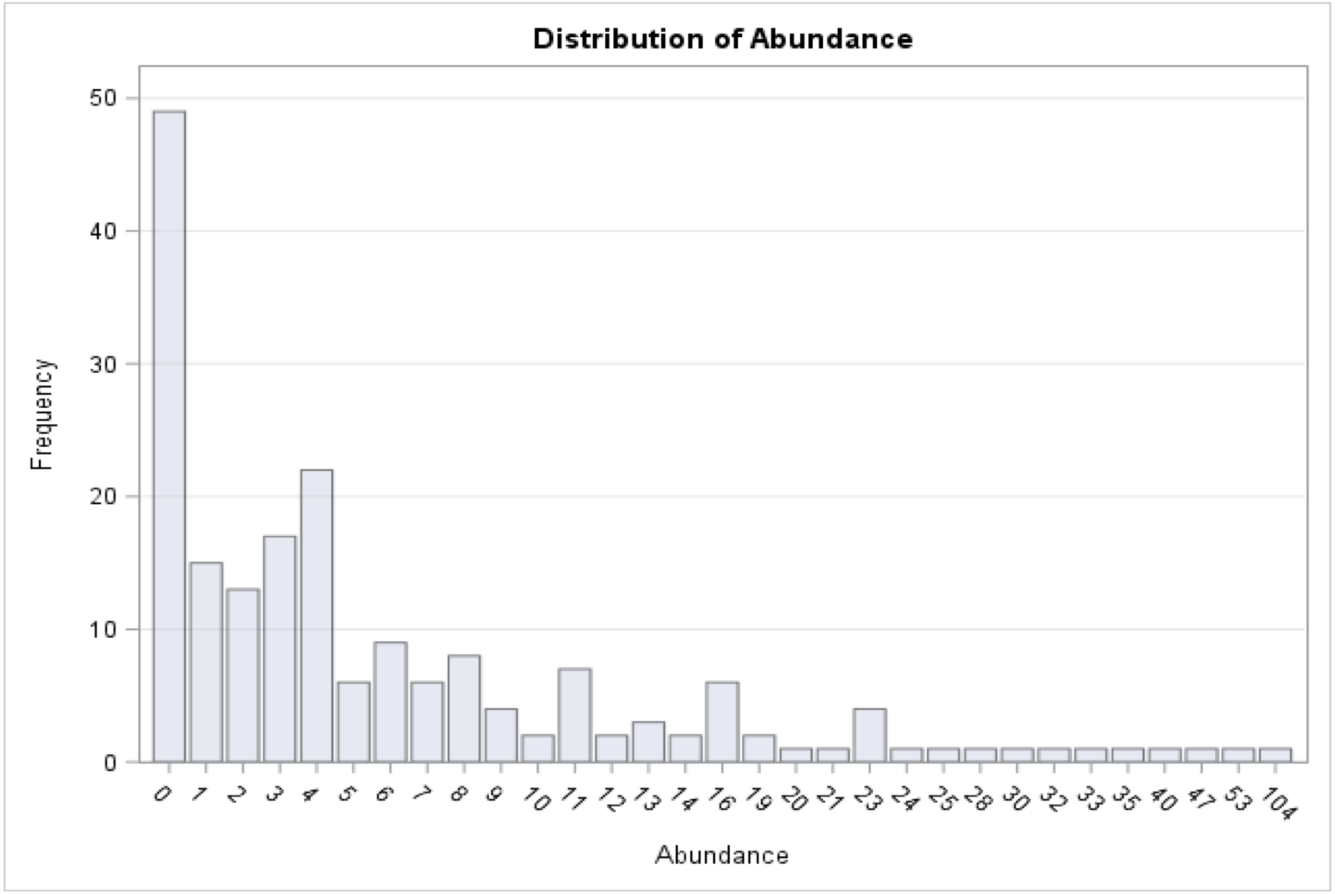
Distribution of medusa abundance. On the *x*-axis is the number of medusa/day, on the *y*-axis the frequency. During 49 of the 190 days of sampling, 0 medusae were found. The maximum number of medusae found in one day is 104. Days with high number of medusae are rare (tail of distribution).

A total of 1,321 individual medusae were collected over 190 sampling days from September 1st, 2015 to September 30th, 2016 (Table 5). Samples were collected an average of 14.7 days per month. The daily average abundance for the sampling period was 6 medusae/day, the median 4, and standard deviation 11.3 (Table 1). The fact that the median is substantially lower than the mean suggests that the distribution of medusa counts is right-skewed, while the fact that the standard deviation is almost twice than the mean suggests that the variation is very high. Figure 3 reports the distribution of medusa counts and confirms the existence of infrequent days with extremely large counts. For instance, the maximum number of medusae found in a day was 104 and occurred on April 11, 2016. Over the 13 sampling months 19 blooms (defined as a day with an abundance at least 1 standard deviation from the mean daily abundance and represented in Fig. 1A by a dashed line) were recorded. The minimum abundance for a bloom was 19 medusae. Five blooms occurred during the summer, and were dominated by species such as *Obelia, Clytia, Malagazzia carolinae, Liriope tetraphila*. Six blooms occurred during the winter, and were dominated by *Liriope tetraphilla, Nemopsis bachei* and *Ectopleura dumortierii.* Eight blooms occurred during the spring and were dominated by *Nemopsis bachei, Liriope tetraphilla* (see Table 2 supplementary material for individual barcoded during each day). 49 out of 190 sampling days showed 0 medusa. The months with the highest medusa abundance were April (248 medusae), February (218), March (197) and September (182 medusae in 2015, and 173 in 2016). The months with the lowest medusa abundance were November (30 medusae), December (1), June (38), July (37) (see Table 5 for details). The presence of blooms, making the distribution of medusa counts right-skewed, has ultimately prompted us to propose several regression specifications with the dependent variable expressed in logarithms.

**Table 5:** Species list and presence per month of sampling period. “?” indicates individual medusae with a best BLAST hit identity value <95%. Their identification is considered dubious (see text for details).

**Species**	**Sep-15**	**Oct-15**	**Nov-15**	**Dec-15**	**Jan-16**	**Feb-16**	**Mar-16**	**Apr-16**	**May-16**	**Jun-16**	**Jul-16**	**Aug-16**	**Sep-16**	**Total #/species**
*Aequorea australis*	2													**2**
*Blackfordia virginica*		1				2	1	2	30	13				**49**
*Bougainvillia muscus*						2								**2**
*Bougainvillia triestina*							1				1			**2**
*Clytia elsaeoswaldae*?													2	**2**
*Clytia folleata*									2				3	**5**
*Clytia gracilis*	3		4				3	17	8	1	1		10	**47**
*Clytia* sp. 1							2	1						**3**
*Corymorpha nutans*						10	12							**22**
*Stauridiosarsia reesi*	1												1	**2**
*Earleria quadrata* ?	12													**12**
*Ectopleura dumortierii*						28	1						6	**35**
*Eucheilota maculata* ?							7	2					3	**12**
*Hydractinia americana*							1							**1**
*Koellikerina fasciculata* ?							1	1						**2**
*Liriope tetraphylla*	1					5	40	128	3		8	8	5	**198**
*Lovenella assimilis* ?	2							1	1	1	1		5	**11**
*Malagazzia carolinae*	12	3	1				9	22	12	3	11	2	13	**88**
*Nemopsis bachei*	5	8	1		12	132	59	19	1					**237**
*Obelia bidentata*	2							1						**3**
*Obelia dichotoma*	16	15	7				3				1		12	**54**
*Obelia geniculata* ?	1		1							5				**7**
*Obelia* spp.	79	30	16				4		1	1	1	11	24	**167**
*Sertularella diaphana*											3			**3**
*Turritopsis dohrnii*										1				**1**
Unknown	46	9	0	1	1	39	53	54	9	13	10	30	89	**354**
**Total #/month**	**182**	**66**	**30**	**1**	**13**	**218**	**197**	**248**	**67**	**38**	**37**	**51**	**173**	**1,321**

### Hydromedusa and environmental factors

Temperature varied from 11.7 °C (min) to 32  °C (max), salinity from 6.00 ppt to 31.97 ppt, and DO from 4.23 µg/L to 10.42. µg/L , chlorophyll a from 0 to 9 µg/L (Table 1). Data for chlorophyll a (µg/L) are available each sampling day from January 2016-September 2016 only (Fig. 1C). As can be seen from Table 3, no significant correlation was found between the number of medusae and temperature. However, inspection of the data suggests that the relation between temperature and abundance is not linear, and a simple correlation measure would fail to capture such non-linearity. Figure 2 reveals that medusa are abundant during periods of mild temperature. Specifically, the bar chart shows that, by dividing the sample into 4 equally-sized temperature quartiles, more medusae were captured during days of intermediate temperatures (the two middle quartiles) relative to days with more extreme weather (first and fourth quartiles), the difference being in the order of a 50% increase. The boxplot in Fig. 2B further explores the distribution of medusa abundance over the same temperature sub-periods and confirms that the median counts (horizontal bar within the boxes, which capture the mid-50% of the distribution) are also higher during the days of mild temperature. Moreover, the boxplots reveal that positive outliers (typically blooms) are more likely during mild temperature days.

The correlation between the other explanatory variables and abundance also seems weak (Table 3), with salinity being marginally statistically significant and remaining variables being insignificant. However, a univariate analysis linking environmental factors to abundance individually is likely deficient because these factors likely interact to produce their outcome on medusa abundance. Moreover, given the high correlation among the explanatory variables, univariate analysis could result in an omitted-variable bias.

To better understand the effect of environmental factors on medusa abundance, multivariate ordinary least squares (OLS) regressions were run using the daily medusa abundance (both in levels and natural logarithms) as the dependent variable, and temperature, salinity, DO, and chlorophyll a as the independent variables. The first 5 models use actual medusa counts as the dependent variable, while the next 5 models use the natural logarithm of counts. To account for potential non-linear effects of temperature on abundance, we either include a quadratic term (Models 2–4, 7–9) or several temperature quartile dummies. As can be seen from the table, models ignoring non-linearity (Model 1 and 6) have a relatively poor model fit, as evidenced by a lower R squared. Moreover, these naive models would lead to the erroneous conclusion that temperature is unrelated to abundance due to lack of statistical significance (high *p*-values). On the other hand, the other models in the table point to a strong and statistically significant non-linear effect of temperature on abundance. For instance, the quadratic model (column 2 in the table) shows that both the linear and quadric term are statistically significant at the 5% level. The interpretation of the coefficient is that for low levels of temperature the linear term (i.e., 2.16) dominates, but the quadratic term (−0.08) becomes more important as temperature rises. These estimates indicate an intermediate level of temperature associated with maximum abundance. Specifically, this level is obtained by solving 2.16–2*0.08*Temperature = 0, which is approximately 21 °C in this case. A somewhat more general approach, one that does not rely on a stringent quadratic assumption, for assessing the impact of temperature is to use several temperature dummies. The estimates in column 3 suggest that, relative to the benchmark case of high temperature, mildly lower and mildly higher temperatures are associated with respectively 8.7 and 6.5 additional individuals per sampling day on average. Considering the fact that on average 5 individuals are collected during hot days (not reported), our estimates represent an average increase of more than 100%.

To account for potential seasonality and unobserved factors driving medusa abundance we also run some regressions (Model 3 and 8) including quarterly time fixed effects (time-varying intercept, *α*_*t*_, in Eq. (1)). The estimates of time fixed effects, which we do no report in the table for brevity, are obtained by including calendar quarter dummies in the regression. As can be seen (Model 3 and 7), the inclusion of time fixed effects increases model fit (higher R squared), but does not drive away the impact of the other regressors (especially temperature) on abundance. This result is important because it helps us rule out with some confidence that the impact of temperature we document is not spurious, or driven entirely by unobserved factors that we might not be accounting for. Finally, while the coefficient estimates on temperature and squared temperature in Model 3 appear to differ from those of Model 2, we note that a similar reasoning used above reveals an approximate level of temperature of 20.5 degrees Celsius associated with maximum abundance. Thus, our finding on temperature are robust across different models.

### Species identification and molecular barcoding

Morphological identification of planktonic hydromedusae is difficult because of their small size, the high number of species, and the morphological disparity through out the life cycle, as new-born medusae and adult medusae may look substantially different. Identifying hundreds of medusae to the species level becomes a time and cost-prohibitive enterprise. To manage species identification we thus adopted a combined approach between morphological identification and molecular barcoding.

After collection and during sorting, medusae (a total of 1,321) were identified at the lower possible level using appropriate keys. Most medusae could be identified to the genus level, some at the family level, only few could be identified at the species level. Specimens were also photographed, and fixed in ethanol for barcoding studies. DNA was then extracted from all the individual medusae and 470 mitochondrial ∼600 bp sequences of the large ribosomal subunit of the mitochondrial RNA (lsu-rRNA, 16S) were generated (See Table S2, for Blast results and Genbank accession number of the newly generated sequences). This represents 36% of all specimens. The relatively low success rate is possibly due to the small size of some of the medusae (i.e., *Obelia*). Each sequence was compared with the online repository Genbank using the BLAST feature and results were used to augment morphological identification. For each query sequence, blastn of the NCBI-BLAST package was run locally, and the result was parsed to retrieve topmost match. We grouped the sequences in 3 categories depending on the level of identity with their best match as follow: identification through Genbank was considered reliable when genetic identity with best match was between 100% to 98% (category 1); it was considered acceptable but with lower confidence, when genetic identity was between 97.9 to 95% (category 2); identification was considered uncertain when genetic identity was lower than 94.9% (category 3). These specimens probably belong to the genus or family of best match. We understand these are arbitrary threshold and need to be taken as an aiding tool to morphological identification. They also can help spotlight the amount of species that are currently missing in Genbank.

Table S2 shows the 470 individual medusa that were successfully sequenced and their BLAST results (best match, % identity between sequences, e-value and query coverage). Using the combined approach between morphological analyses and barcoding we identified 967 individual medusae as belonging to 25 species (see Table 5 for a complete list). Of this 11 species, or 44% of total (*Aequorea victoria, Blackfordia virginica, Clytia folleata, Clytia gracilis, Malagazzia carolinae, Nemopsis bachei, Obelia bidentata, Obelia dichotoma, Turritopsis dohrnii* and *Sertularella diaphana*) were in category 1 (identified with confidence), 6 (24%) *(Bougainvillia triestina, Bougainvillia muscus, Stauridiosarsia reesi, Ectopleura dumortierii, Hydractinia americana, Liriope tetraphylla)* in category 2 (identified with lower confidence), and 8 (32%) in category 3 (uncertain identification). These were mostly rare species found only once and with relatively low number of individuals (their best match in Genbank was *Clytia elsaeoswaldae*, *Corymorpha nutans*, *Clytia* sp. 1*, Earleria quadrata, Eucheilota maculata, Koellikerina fasciculata, Lovenella assimilis, Obelia geniculata*). 354 individual medusae for which barcoding sequence was not available and that could not be identified morphologically (because undescriptive juvenile or damaged medusa), remain unidentified (see Table 5). Sequences are in Genbank under the following accession numbers: MN364418 –MN364461, MN355950 –MN355952, MN355770 –MN355845, MN355846 –MN355904, MN355575 –MN355691, MN355905 –MN355911, MN355912 –MN355949, MN355718 –MN355768, MN355692 –MN355717, MN355508 –MN355516, MN347011 –MN347014, MN343754 –MN343758, MN341844 –MN341854 and MN367046 –MN367055.

### The community composition and seasonality of dominant species of hydromedusa

**Figure 4:**
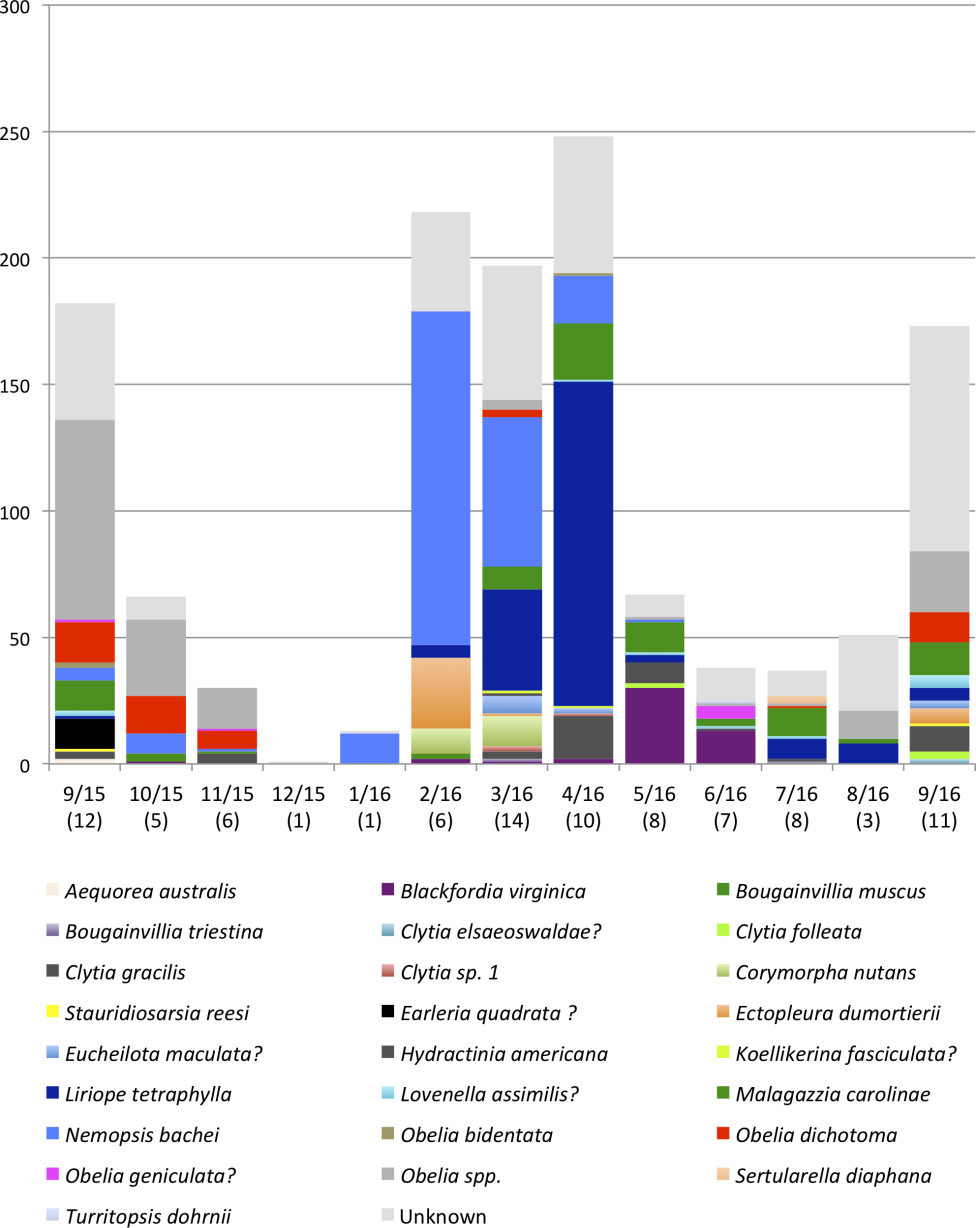
Monthly medusa abundance and species richness. On the *x*-axis are sampling month and number of species (in brackets). On the *y*-axis are numbers of individual medusae collected during the month. Color blocks indicate species.

*Malagazzia carolinae* was the most common species, found in 10 out of the 13 sampled months. Several species including *Aequorea australis, Bougainvillia muscus, Clytia elsaeoswaldae,* and *Turritopsis dohrnii* were found only once during the sampling period. Many *Obelia* medusae for which barcoding was not successful, were morphologically identified to the genus level (due to their distinctive generic characters) but could not be identified at the species level, and were all collectively called *Obelia* spp. It is possible that this category contains more than one *Obelia* species. Table 6 shows the dominant species for each month, and Table 7 shows the diversity index (Shannon Weaver) per month. Only one medusa was found during December 2015 and could not be identified to species, so it is not included in the dominance analysis. The genus *Obelia* dominated the months September 2015–November 2015 and August 2016–September 2016. *Nemopsis bachei* was the dominant species January 2016–March 2016, *Liriope tetraphylla* was the dominant species in April 2016. The dominant species in May 2016 was *Blackfordia virginica*. *Malagazzia carolinae* dominated June 2016–July 2016. March, July and September were the months with higher diversity index (Table 7). March 2016 had the highest number of species (14) followed by July and September 2016 (11 each). December 2015 and January 2016 had only one species (Fig. 4 and Table 5).

**Table 6:** Top two dominant species for each month of sampling period.

**Month**	**Species**	**Dominance**
Sep-15	*Obelia* sp.	0.3005
	*Malagazzia carolinae*	0.0507
Oct-15	*Obelia* sp.	0.3147
	*Obelia dichotoma*	0.1049
Nov-15	*Obelia* sp.	0.3692
	*Obelia dichotoma*	0.1077
Jan-16	*Nemopsis bachei*	0.5680
Feb-16	*Nemopsis bachei*	0.3726
	*Ectopleura dumortierii*	0.0296
Mar-16	*Nemopsis bachei*	0.1843
	*Liriope tetraphylla*	0.1250
Apr-16	*Liriope tetraphylla*	0.3176
	*Malagazzia carolinae*	0.0682
May-16	*Blackfordia virginica*	0.2067
	*Malagazzia carolinae*	0.1378
Jun-16	*Malagazzia carolinae*	0.0607
	*Obelia geniculata*	0.0304
Jul-16	*Malagazzia carolinae*	0.2287
	*Liriope tetraphylla*	0.1331
Aug-16	*Obelia* sp.	0.1493
	*Liriope tetraphylla*	0.0965
Sep-16	*Obelia* sp.	0.0960
	*Malagazzia carolinae*	0.0578

**Table 7:** Shannon weaver index. The higher the Shannon Weaver index, the more diversity. The three lowest values are colored in blue, and the three highest in grey.

Month	Shannon Weaver
Sep-15	1.68310
Oct-15	1.42658
Nov-15	1.28360
Jan-16	0.27119
Feb-16	1.18928
Mar-16	1.85532
Apr-16	1.43541
May-16	1.62339
Jun-16	1.58412
Jul-16	1.73702
Aug-16	1.06056
Sep-16	1.70373

### Relationship between dominant species and environmental factors

**Figure 5:**
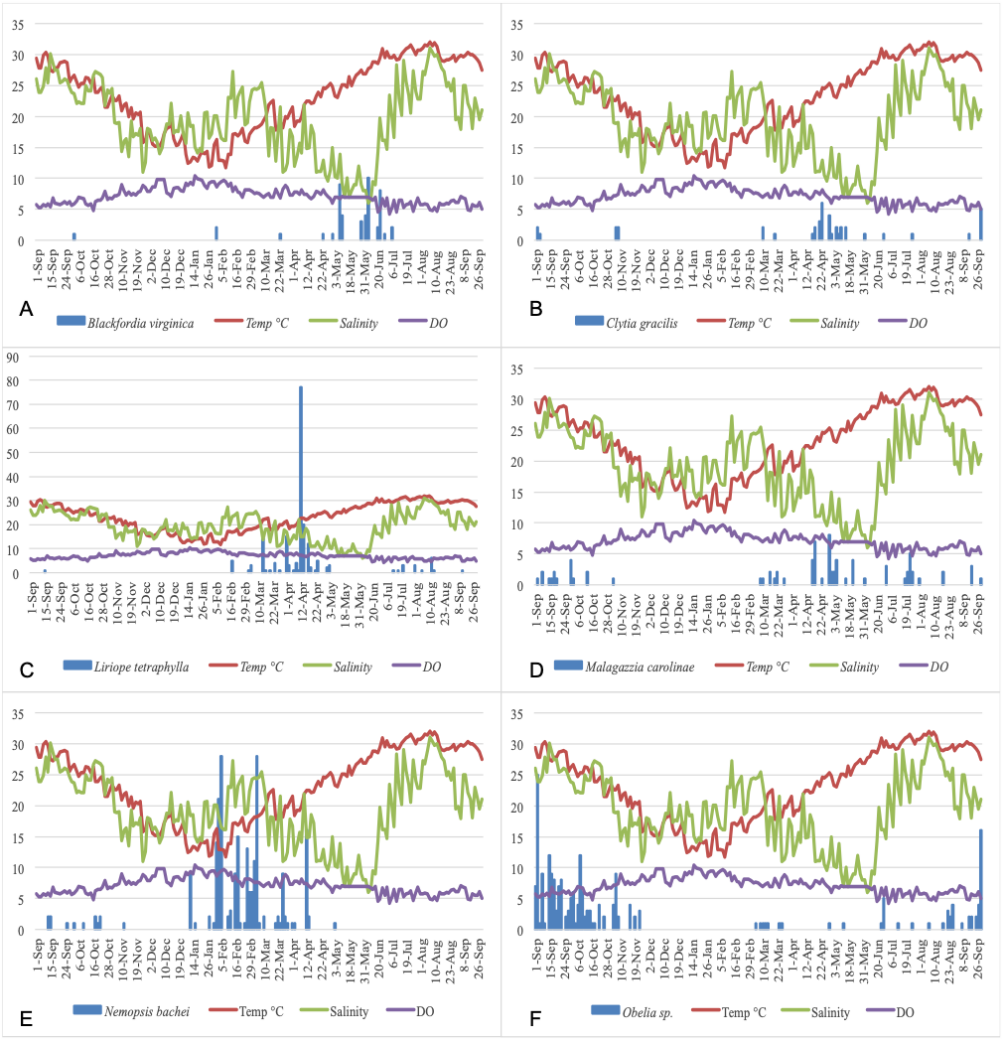
Relationship of the five most abundant taxa with temperature (°C), salinity (ppt), and DO (mg/L). (A) *Blackfordia virginica.* (B) *Clytia gracilis.* (C) *Liriope tetraphylla.* (D) *Malagazzia carolinae.* (E) *Nemopsis bachei.* (F) *Obelia* spp.

Figure 5 show the plots of each species abundance with temperature, salinity, and DO and suggests that each species has a distinct seasonality. Regression models were run for the top five dominant species (*Blackfordia virginica*, *Clytia gracilis, Liriope tetraphylla, Malagazzia carolinae, Nemopsis bachei*)*,* and the genus *Obelia,* to determine their specific relationships with environmental factors. The species-specific models were run using medusa abundance as the dependent variable, temperature (linear and non-linear), salinity, and dissolved oxygen as the independent variables, and time fixed effects classified by season (Table 8). This corresponds to Model 3 in total medusa count (see above and Table 4). Model 3 was chosen because it incorporates linear and non-linear temperature regression and time fixed effect. Under this model, temperature had a significant effect on the abundance of *Clytia gracilis, Liriope tetraphylla, Malagazzia carolinae,* and *Obelia*. Salinity and DO, but not temperature, had a significant effect on the abundance of *Nemopsis bachei*.

**Table 8:** Best-fit regression models. Best-fit regression models run to find the relationship between dominant species and environmental factors. *P*-values are presented in parenthesis underneath their corresponding coefficients. Values significant at alpha = 0.05 are highlighted in bold.

	*Blackfordia virginica*	*Clytia gracilis*	*Liriope tetraphylla*	*Malagazzia carolinae*	*Nemopsis bachei*	*Obelia* spp.
Temperature	−0.085091	0.2967857	1.335539	0.3557258	0.2277842	1.265557
	(0.4929)	(0.0007)	(0.0216)	(0.0268)	(0.7171)	(0.0005)
Temperature^2^	0.0029645	−0.0070505	−0.037724	−0.0081399	−0.0083639	−0.028353
	(0.3956)	(0.0012)	(0.0301)	(0.0514)	(0.5319)	(0.0019)
Salinity	−0.0248638	−0.0177534	0.389765	−0.012308	0.2161154	0.061861
	(0.1388)	(0.1768)	(0.1904)	(0.6265)	(0.0069)	(0.1464)
DO	−0.0016194	−0.0008594	0.016066	−0.002049	−0.0359073	0.007388
	(0.0551)	(0.5489)	(0.4022)	(0.4096)	(0.0123)	(0.1842)
Fixed time effects	Yes	Yes	Yes	Yes	Yes	Yes
Number of observations	191	191	191	191	191	191
R-Square	0.1266	0.1197	0.1247	0.1039	0.2288	0.178

## Discussion

### Environmental variables

Temperature has a non-linear relationship with medusa abundance (Table 4). Using regression analysis based on a quadratic model (Table 4), we have identified a level of temperature of approximately 21 °C that is associated with maximum medusa abundance in Galveston Bay. Regression analysis using temperature quartiles as dummies produces finding consistent with the quadratic model: the highest total medusa abundance is found during days with moderate temperature (medium low and medium high temperature), and decreases sharply otherwise (Figs. 2A and 2B). Temperature also shows a strong effect on the abundance of many of the dominant species (Table 8). This is consistent with previous studies conducted on individual species of Hydrozoa (Ma & Purcell, 2005; Nowaczyk et al., 2016; Wintzer, Meek & Moyle, 2013). Salinity was not significantly correlated with total medusa abundance, however *Nemopsis bachei* showed seasonality and had a significant relationship with salinity and DO (Fig. 5). This is consistent with previous studies on this species, which have shown a correlation with salinity and not temperature (Nowaczyk et al., 2016). *Blackfordia virginica* was also one of the dominant species during spring but was not statistically correlated with any of the tested environmental factors (Table 8). These individual species analyses and the fact that dominant monthly species changed with time (Table 6) indicate that trigger of medusa production may be species-specific (Nowaczyk et al., 2016; Wintzer, Meek & Moyle, 2013).

### Gulf of Mexico and Galveston Bay Hydrozoa diversity

The most recent species list of Hydrozoa for the Gulf of Mexico was compiled by Calder & Cairns (2009) and listed 214 species. Only eight of the species recorded in this study were on the Calder & Cairns checklist. An additional seven species had been reported in the GoM in previous studies (See Table 9). In total, eight species found in this study had never been recorded in the GoM before. The most recent study on Hydrozoa in Galveston Bay was conducted by Defenbaugh and Hopkins in 1973. They surveyed only polyps and found 25 species. Only 4 of the 25 species found in this study were previously described in Defenbaugh and Hopkins (Table 9). Thus 21 species are recorded for the first time in the Galveston Bay.

**Table 9:** References for species previously recorded in the Galveston Bay and in the Gulf of Mexico.

**Species**	**Galveston Bay**	**Gulf of Mexico**	**Reference**
*Aequorea australis*			
*Blackfordia virginica*		X	Cairns & Fautin (2009), Harrison, Kim, & Collins (2013)
*Bougainvillia muscus*		X	Calder & Cairns (2009)
*Bougainvillia triestina*			
*Clytia elsaeoswaldae^a^*			
*Clytia folleata*		X	Cairns & Fautin (2009)
*Clytia gracilis*	X	X	Defenbaugh & Hopkins (1973), Calder & Cairns (2009)
*Clytia* sp. 1			
*Corymorpha nutans*		X	Cairns & Fautin (2009)
*Stauridiosarsia reesi*			
*Earleria quadrata^a^*			
*Ectopleura dumortierii*		X	Calder & Cairns (2009)
*Eucheilota maculata^a^*			
*Hydractinia americana*		X	Calder & Cairns (2009)
*Koellikerina fasciculata^a^*		X	Martell-Hernández, Sánchez-Ramírez & Ocaña Luna (2014)
*Liriope tetraphylla*		X	Cairns & Fautin (2009)
*Lovenella assimilis^a^*			
*Malagazzia carolinae*		X	Mayer (1900)
*Nemopsis bachei*		X	Cairns & Fautin (2009)
*Obelia bidentata*	X	X	Defenbaugh & Hopkins (1973), Calder & Cairns (2009)
*Obelia dichotoma*	X	X	Defenbaugh & Hopkins (1973), Calder & Cairns (2009)
*Obelia geniculata^a^*	X	X	Defenbaugh & Hopkins (1973), Calder & Cairns (2009)
*Obelia* spp.			
*Sertularella diaphana*		X	Allman & de Pourtalès (1877), Calder & Cairns (2009)
*Turritopsis dohrnii*		X	Miglietta, Maggioni & Matsumoto (2018)

**Notes.**

a indicates species in category 3 (identity with blast top hit <95%).

Ours study focuses on the medusa stage only, thus neglecting Hydrozoa that have lost the medusa stage (with the exception of one species *Sertularella diaphana*, that was caught in our plankton tows in the form of sporosacs). This may explain some of the discrepancies between the species found in our sampling and those found in other published studies that focused only on benthic sampling. It is also noteworthy that the Galveston Bay area has a large amount of ship traffic exposing it to potential species introduction through ballast water, which may dramatically alter its species diversity and composition through time (Steichen et al., 2012). The majority of the dominant species (with the exception of *Malagazzia carolinae*) found during this research are however widely distributed throughout the GoM (namely *Blackfordia virginica, Clytia gracilis, Liriope tetraphylla, Nemopsis bakei*) (see Table 9 for references). In contrast, the dominant *Malagazzia carolinae* was first described in 1900 by Mayer in Tortugas, Florida, an island at the edge of the Gulf of Mexico, and never recorded in the GoM. *M. carolinae* is generally found on the coasts of New Zealand and China (Bouillon, 1995; Du et al., 2011). More surveys will be required before we can know the extent of the distribution and persistence of *Malagazzia carolinae* in the GoM. *Aequorea australis, Bougainvillia triestina, Stauridiosarsia reesi,* are new to the GoM. The introduced *Turritopsis dohrnii* (Miglietta & Lessios, 2009; Miglietta, Maggioni & Matsumoto, 2018) was recently described for the first time in the GoM. All of these species had BLAST identity above 95% (Table S2), but had sample sizes lower than 5 individuals. Other species with low BLAST identity (thus with uncertain identification) were found for the first time in the GoM , and their BLAST top hits were *Clytia elsaeoswaldae*, *Earleria quadrata*, *Eucheilota maculata*, *Koellikerina fasciculata*, and *Lovenella assimilis* and *Obelia* spp. Those are rare and/or understudied species, as shown by the lack of high identity matches on Genbank. More data from the GoM and worldwide and in-depth morphological identification are needed in order to further discuss the implications of these findings. Our results however indicate that the Hydrozoa in this region of the world are still heavily underestimated. They also show that taxonomic studies that couple molecular barcoding and morphological identification, and studies that focus on the full life cycle of hydrozoan species (polyp and medusa) are needed to assess biodiversity. Monitoring local biodiversity and its long-term changes due to human disturbance is particularly important in high traffic marine areas such as Galveston Bay and the GoM in general.

## Conclusion

The hydromedusae of Galveston Bay were collected over a 13-month period and identified through a hybrid approach between morphological and molecular techniques to assess the species diversity and abundance. We found 25 species, 21 of which were never previously recorded in the Galveston Bay, and eight never recorded in the Gulf of Mexico. The daily hydromedusa abundance was compared to temperature, salinity, DO, and chlorophyll a through multiple multivariate OLS regression models. The models suggest that temperature has a non-linear relationship with medusa abundance and is statistically correlated with medusa abundance in the field. More specifically the highest medusa abundance was recorded when the temperature was at intermediate values, and the lowest medusa abundance was recorded during the hottest quartile. This has interesting implication on future scenario for global warming, as it is expected that the temperature in the GoM will rise by 4 °C by the end of the century (Muhling et al., 2011; Biasutti et al., 2012). Our results indicate that total medusa abundance may decrease in response to elevated/extreme temperature.

When looking at single dominant species, we show that temperature has a significant effect on the abundance of *Clytia gracilis, Liriope tetraphylla, Malagazzia carolinae,* and *Obelia* and that salinity and DO, but not temperature, have a significant effect on the abundance of *Nemopsis bachei*.

In summary, our results suggest that there is seasonal fluctuation in the abundance and diversity of hydromedusa in Galveston Bay that is partially driven by temperature. It also shows different species produce medusa in response to different environmental cues, such as temperature or salinity and DO. This study represents the first look into the hydromedusa community of Galveston Bay. Further studies and long-term monitoring are necessary to broaden our understanding of plankton dynamics and its drivers, and assess the diversity of Hydrozoa in the GoM.

##  Supplemental Information

10.7717/peerj.7848/supp-1 Supplemental Information 1 Supplementary Tables 1 and 2 Supplementary Table 1: Raw data showing date, medusa count, temperature, salinity, dissolved oxygen (DO), and chlorophyll a
Supplementary Table 2: Sequences produced in this paper with their BLAST tophit resutls, Identity %, query % coverage, and *e*-values. Newly produed sequences have been submitted to Genbank with the following accession numbers: MN364418 –MN364461, MN355950 –MN355952, MN355770 –MN355845, MN355846 –MN355904, MN355575 –MN355691, MN355905 –MN355911, MN355912 –MN355949, MN355718 –MN355768, MN355692 –MN355717, MN355508 –MN355516, MN347011 –MN347014, MN343754 –MN343758, MN341844 –MN341854 and MN367046 –MN367055.

10.7717/peerj.7848/supp-2 Supplemental Information 2 Fasta File with Sequences The names of the sequences represent collection date and unique number associated with each medusa collected in this study.

## Additional Information and Declarations

### DNA Deposition

The following information was supplied regarding the deposition of DNA sequences: Sequences are attached in FASTA format. All sequences are available at Genbank: MN364418 –MN364461, MN355950 –MN355952, MN355770 –MN355845, MN355846 –MN355904, MN355575 –MN355691, MN355905 –MN355911, MN355912 –MN355949, MN355718 –MN355768, MN355692 –MN355717, MN355508 –MN355516, MN347011 –MN347014, MN343754 –MN343758, MN341844 –MN341854 and MN367046 –MN367055.

### Data Availability

The following information was supplied regarding data availability: All raw data are available in the Supplemental File.
